# Screening for Risk of Fall-Related Inpatient Trauma in a US Acute Care Setting

**DOI:** 10.7759/cureus.63199

**Published:** 2024-06-26

**Authors:** Barbara Ragione, Lauren Rothburd, Theresa Drucker, Sarah Eckardt, Patricia A Eckardt

**Affiliations:** 1 Nursing Quality Improvement, Good Samaritan University Hospital, West Islip, USA; 2 Trauma, Good Samaritan University Hospital, West Islip, USA; 3 Nursing, Molloy University, Rockville Centre, USA; 4 Process Improvement, Northwell Health, Huntington, USA; 5 Nursing, Good Samaritan University Hospital, West Islip, USA

**Keywords:** falls, cerebrovascular accidents, falls efficacy scale, morse fall scale, fall prevention

## Abstract

Introduction

Falls during hospitalization are a leading cause of preventable trauma-related injuries. Factors associated with fall risk include an unfamiliar environment, changes in health status, and efficacy based on the home environment. Assessing fall efficacy with an individualized prevention plan can decrease falls. The primary aim of this study was to estimate the effect of implementing a fall efficacy screening and intervention on reducing patient falls.

Methods

The study utilized a quasi-experimental, cross-sectional design with a convenience sample of patients admitted to an in-patient adult medical unit within a community hospital over a twelve-month period. Sampling times included pre-implementation, immediately post-implementation, and a second post-implementation phase. The intervention consisted of an admission fall efficacy screening tool and an individualized educational initiative. Statistical analysis included descriptive statistics of central tendency and dispersion, along with inferential statistics using independent sample t-tests, chi-square tests, correlations, and binary logistic regression.

Results

Among the study participants (n=2,074), the total sample had an average age of 67.7 (+/- 17.4) years and had mean scores of 13.3 (6.9) on the Short Falls Efficacy Scale-International and 51.8 (20.3) on the Morse Fall Scale. Fifty-two percent of the study population were female; 16.2% of the patients were diagnosed with cerebrovascular accident (CVA) or CVA-like symptoms. Fall rates decreased with a rate of change of -4.15% after efficacy screening and intervention. Males demonstrated higher efficacy in avoiding falls compared to females (t(828) = 3.369, p <0.001). Patients with a CVA diagnosis demonstrated higher efficacy scores compared to non-CVA patients (t(2071) = -3.348, p <0.001). FES risk groups (OR of 5.632, 95% CI (2.171-7.892)) and age over 65 (OR 1.21, 95% CI (1.006-1.442)) were significant predictors of a fall when patients with a primary CVA diagnosis were omitted from the sample (p= 0.022 and 0.046 respectively).

Conclusion

The findings suggest that efficacy screening may be associated with decreased falls for acute care non-CVA inpatient populations over 65 years of age. Further research into the predictive utility of fall efficacy screening in acute care CVA and non-CVA hospitalized patient populations aged 65 years and above is recommended.

## Introduction

Each year, between 700,000 and 1,000,000 people in the United States fall in the hospital [1]. Although falls are preventable, patient falls have become the most common adverse event experienced by patients in acute care facilities and continue to challenge overall healthcare quality [1]. Fall prevention is a priority for most institutions; however, the implementation of fall risk screening assessments and fall prevention programs often does not decrease patient falls [2]. Most fall assessment tools are nurse-driven and identify physiological factors contributing to fall risk but do not assess perception of risk [3]. Patients may overestimate their control over fall prevention, thus increasing their risk of falling and associated trauma during their hospitalization. Specific patient subpopulations, including male patients and patients diagnosed with a cerebral vascular accident (CVA), have been found to have an altered higher perceived efficacy regarding their ability to prevent falls, posing a higher risk for falls [4, 5, 6]. Unlike fall screening practices in community settings, the common fall risk screening tools used in acute care settings do not screen for fall efficacy [3, 7].

There are multiple psychometrically validated fall assessment tools, including the John Hopkins Fall Risk Assessment Tool (JHFRAT), Morse Fall Scale (MFS), Hendrich II, Stratify, and Tinetti scales, used in acute care settings today. A recent meta-analysis found that due to the heterogeneity of sensitivity and specificity in predicting falls, none of the commonly used physiological-centric fall risk assessment tools can be recommended as the standard in acute patient settings [8]. Strategies to prevent patient falls require a patient-centered approach with patients engaged in the risk assessment and prevention plan [9, 10].

The Fall Efficacy Scale (FES), though widely used in community and long-term care settings [11, 12], is currently not a standard tool used within the acute care setting [13, 14]. The FES asks patients specific questions regarding their ability to complete activities with or without assistance, such as "how confident are you that you can get dressed without assistance" [13, 14]. Gazibara T et al. (2017) found the FES to be a predictor of patients who may experience multiple falls [15]. However, Greenberg M et al. (2021) did not observe a statistically significant difference in median FES scores among patients who experienced a fall compared to those who did not (11 vs. 10; p=0.12) [16]. Gettens S et al. (2015) concluded that a high FES score could also be a predictor of fall risk [17]. Patients who demonstrate an inaccurate perception of fall risk should receive appropriate education about why they are at risk for falls [17]. Additionally, Kuhlenschmidt ML et al. (2016) found that patient education tailored to the patient’s risk could help the patient genuinely understand why they are at risk; they found that after the education, the patient’s perception of risk was appropriately assessed by the patient [18].

Albert Bandura’s theory of self-efficacy serves as the underlying foundation for assessing the implementation of a fall risk tool that evaluates a patient’s perception of his or her ability. Bandura’s self-efficacy theory (SET) conveys a person’s perception of his or her own ability to achieve a goal [19]. The theory was founded on the principal belief that psychological perceptions function as a means of developing and strengthening expectations of personal efficacy [20]. Additionally, applying the SET theoretical foundation can assist in evaluating the patient’s understanding of their perceived abilities and their adaptation to change during an acute hospitalization.

Therefore, the primary aim of this study was to estimate the effect of implementing a patient fall efficacy screening tool on patient fall rates. This study examined the differences in fall efficacy between specific patient subpopulations hypothesized to have falsely high fall prevention self-efficacy. Additionally, the relationship between scores on fall efficacy and standard fall risk screening tools for patient subpopulations hypothesized to have an inverse relationship between these measures was explored.

## Materials and methods

Study design

This was a quasi-experimental, cross-sectional design using a convenience sample. The setting was a Level 3 Trauma Center in Southeast New York for patients admitted to an in-patient adult medical unit during a 12-month period (January 2019 to December 2019). Sampling included three time points: pre-implementation, immediately post-implementation, and second post-implementation. The intervention was the implementation of the Short FES-I as an admission screening tool and educational intervention on the convenience sample of all patients meeting the inclusion criteria.

Protection of Human Subjects and Ethical Conduct of Research

This research activity involving human subjects met the criteria for Exempt Category 4(iii) (2018 Requirements): The research involved only information collection and analysis involving the investigator's use of identifiable health information when that use is regulated under HIPAA [45 CFR parts 160 and 164, subparts A and E, for the purposes of "health care operations" or "research" as those terms are defined at 45 CFR 164.501 or for "public health activities and purposes" as described under 45 CFR 164.512(b)] [21].

Population and Setting

**Figure 1:**
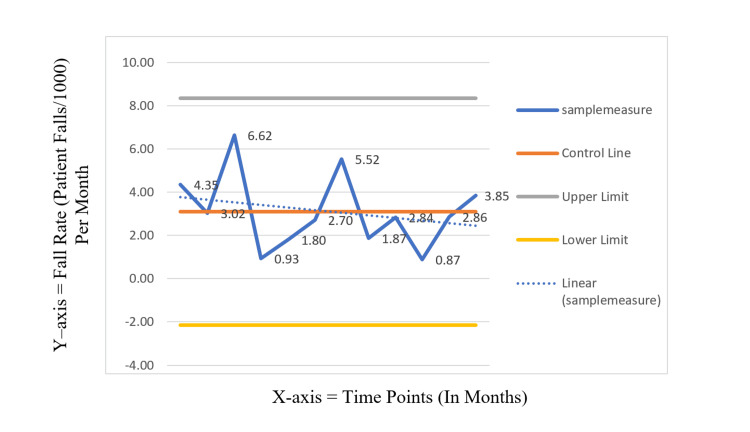
Control chart of fall rate across time, January to December 2019.

The facility setting for this research was a 537-bed (including 100 nursing home beds), non-profit, faith-based medical center located in a suburban setting in the northeast United States serving a geographic catchment area of more than 850,000 individuals. The adult medical unit selected for this study has an approximate census of 40 patients per day with an average length of stay of four to five days. The power calculation was estimated using the Altman nomogram and the standardized difference calculation, based on the prior standard deviation of Short FES-I in similar populations and a clinically meaningful difference between a low-risk FES score and a high-risk FES score to achieve a power of 0.80 [22, 23]. To obtain a power of 0.80 for two-tailed significance testing with a chosen alpha of 0.05 for proportion comparisons of discrete outcomes (3 degrees of freedom for chi-square estimates) and mean differences for continuous outcomes resulted in a minimum sufficient sample size of n = 121 in each group and n = 64 in each group for proportion and mean difference comparisons, respectively [23, 24]. A sample of 524 patients met inclusion criteria and were in the first post-implementation intervention phase. The unit is a mixed-gender unit, with the most common admitting diagnosis of CVA or a similarly related symptomatic diagnosis. Retrospective data of 464 patients from the eight-week pre-implementation period and 1,086 patients from the 24-week second post-implementation period were included for comparison to the first post-implementation sample (n = 524) regarding changes in fall rate over time (Figure 1).

Inclusion criteria

Inclusion criteria were adult patients admitted to an adult medical unit who, during the study’s eight-week intervention phase, had the cognitive ability to answer the questions within the Short FES-I.

Exclusion criteria

Exclusion criteria were adult patients admitted to the unit who, during the study’s eight-week intervention phase, did not have the cognitive ability to answer the questions within the Short FES-I.

Data collection

Data from the electronic health record were abstracted into a double password-protected and encrypted Microsoft Excel workbook to provide comparative data from pre- and post-implementation. Data extraction included fields with the medical record number (MRN) accessed to have a unique identifier for patient subject-level data and date of admission to provide the correct sampling frame.

Short FES-I

The Short FES-I is a seven-item, valid, and reliable instrument currently used for assessing fall risk. Scores for the Short FES-I range from a minimum of 7 (no concern about falling) to a maximum of 28 (severe concern about falling). Literature shows that the FES is a valid tool to identify patients at risk for falls [25]. The FES has demonstrated excellent internal validity (α = 0.96), good relative reliability (intra-class correlation = 0.88), and internal consistency reliability (Cronbach's alpha = 0.94). Additionally, the value for absolute reliability was estimated with a standard error of measure of 2.9 (10.6%), and the smallest real difference was 7.9 (29%) [3, 14, 26]. Following the scoring rules for the Short FES-I, if more than two items are missing on an assessment, the data will not be used for that patient [26].

Morse fall scale

The MFS is a rapid and simple method for assessing a patient’s likelihood of falling. The MFS measures six main variables: history of falling, secondary diagnosis, ambulatory aid, IV or IV access, gait, and mental status. Total scores range from 0 to 125, with 45 as the recommended cut-off score [3]. The MFS demonstrated high clinical reliability and validity upon development, scoring 0.96 in reliability, 0.78 in sensitivity, and 0.83 in specificity. Previous studies have reported a sensitivity of 68% to 84%, specificity of 29% to 83%, and inter-rater reliability of 80% to 96% [8]. Baseline data showed that the average MFS for patients admitted to the facility was 65. Morse defines a score of 45 or higher as the highest risk for falls.

Intervention

Nurses completed the Short FES-I tool in addition to the existing MFS to assess the patient’s risk of falling during the eight-week intervention period. When the risk was identified, nursing staff provided patient-specific education and applied signage to directed areas of the patient environment.

Statistical analysis

Data analyses included descriptive and correlational comparisons and significance testing of hypotheses with percentage comparisons of pre- and post-data for descriptive statistics; a two-tailed chi-squared statistic was estimated for inferential comparison of the proportion of falls pre- and post-implementation. Pearson’s correlation was used to estimate the correlation between the Short FES-I and the MFS for convergent validity. A two-tailed independent-samples t-test was then conducted to compare baseline fall rates and post-implementation fall rates. Fall rates across all three data collection periods were compared over time by changes in fall rates. Differences in hypothesized subpopulations’ fall efficacy scores were compared to other subpopulations using two-tailed independent-samples t-tests for continuous outcomes, and chi-square tests for discrete outcomes. Additionally, a binomial logistic regression model was estimated to predict the probability of falls within the sampled population. The analysis was completed using IBM SPSS Statistics for Windows, version 28 (IBM Corp., Armonk, NY, USA), and Stata Statistical Software: Release 17 (College Station, TX: StataCorp LLC).

## Results

Sample characteristics

There were a total of 2,074 patients in the sample, with an average age of 67.7 (+/- 17.4) years. The total sample comprised 52.0% females, and 16.2% of the patients were diagnosed with CVA or CVA-like symptoms. There were 464 patients in the pre-implementation sample, 514 patients in the first post-implementation sample, and 1,086 patients comprised the second post-implementation sample. Additionally, no differences were found in average age or gender between the three sampling frames. Overall, the sample had a mean Short FES-I score of 13.3 (+/- 6.9) and an MFS score of 51.8 (+/- 20.3) (Table 1).

**Table 1:** Sample demographics by pre-implementation, post-implementation, and second post-implementation sampling frames (n=2,074). FES: Fall Efficacy Scale; MFS: Morse Fall Scale.

Demographic				
	Pre	Post	2nd Post	Total
Age (in years)				
21-30 years	12 (2.59%)	15 (2.86%)	37 (3.41%)	64
31-40 years	26 (5.6%)	25 (4.77%)	70 (6.45%)	121
41-50 years	22 (4.74%)	49 (9.35%)	73 (6.72%)	144
51-60 years	75 (16.16%)	98 (18.7%)	175 (16.11%)	348
61-70 years	94 (20.26%)	92 (17.56%)	215 (19.8%)	401
71-80 years	84 (18.1%)	108 (20.61%)	236 (21.73%)	428
81-90 years	109 (23.49%)	104 (19.85%)	202 (18.6%)	415
91+ years	42 (9.05%)	33 (6.3%)	78 (7.18%)	153
Gender				
Female	250 (53.9%)	272 (51.9%)	556 (51.2%)	1078
Male	214 (46.1%)	252 (48.1%)	530 (48.8%)	996
MFS				
No	31 (6.7%)	39 (7.4%)	62 (5.7%)	132
Low	233 (50.2%)	257 (49.0%)	542 (50.0%)	1032
High	200 (43.1%)	228 (43.5%)	481 (43.8%)	909
FES				
Low	NA	128 (49.8%)	268 (46.9%)	396
High	NA	129 (50.2%)	304 (53.1%)	433
Patient Fall				
No fall	456 (98.3%)	518 (98.9%)	1076 (99.1%)	2050
Fall	8 (1.7%)	6 (1.1%)	10 (0.9%)	24

Instrument reliability estimates with this sample

The Short FES-I was found to be reliable in this sample (α = 0.88). The MFS demonstrated sufficient inter-rater reliability (intraclass correlation coefficients (ICC) = 0.91). The MFS and FES-I were significantly positively correlated for the post-implementation samples (n=1,591) (r = 0.407, n = 14, p < 0.01) (Table 2).

**Table 2:** Reliability and validity estimates of FES and MFS. FES reliability was estimated with Cronbach’s alpha statistic. MFS reliability was estimated with the intraclass correlation coefficient. Convergent validity of the FES and MFS was estimated with Pearson’s correlation. FES: Fall Efficacy Scale; MFS: Morse Fall Scale.

Scale	Items	Reliability Estimate
Fall Efficacy Scale	7	0.88
Morse Fall Scale	6	0.91
Fall Efficacy Scale and Morse Fall Scale	13	0.41

Primary aim

Data from the pre-implementation phase indicated a fall rate of 4.35 in January 2019. In the post-implementation phase, in August 2019, the rate decreased to 1.87. Sustainability was observed through December 2019 with a rate of 3.85, indicating a rate of change of -4.15%. When analyzing the data quarterly, in the first quarter of 2019, the fall rate was 4.69, which decreased to 2.48 in the fourth quarter of 2019.

Secondary aim

Males showed a statistically significant lower FES than females, indicating that males felt more efficacious in fall prevention than females, t (828) = 3.37, p < 0.001 (Table 3).

**Table 3:** Independent t-tests for MFS, FES scores, and gender (n=2,074). **p < 0.01. MFS: Morse Fall Score; FES: Fall Efficacy Scale.

Measure	t	df	Sig. (2-tailed)		Mean Difference	95% Confidence Interval of the Difference	
						Lower	Upper
MFS	1.269	2071		.204	1.131	-.616	2.879
FES	3.369	828		.001**	1.600	.668	2.532

Most males indicated a “low” FES score, compared to females who generally rated themselves as “high” (X² (1, n = 829) = 10.02, p = 0.002) (Table 4).

**Table 4:** Association of sex and fall efficacy scale group (n=829). **p < 0.01.

Measure	*X *^2^	df	Sig. (2-tailed)
Sex	10.02	1	0.002**

Although females accounted for most falls during the study (n=16), there was no statistically significant difference between falls among females and males (X² (1, n = 1594) = 2.09, p = 0.147) (Table 5).

**Table 5:** Association of sex and falls (n=1594).

Measure	*X*^ 2^	df	Sig. (2-tailed)
Sex	2.09	1	0.147

Patients who had a CVA diagnosis indicated statistically significant higher MFS and FES scores. When testing for patients with a CVA diagnosis and their MFS grouping, X² (2, n = 2073) = 15.843, p < 0.001. FES groups indicated a statistically significant difference, X² (1, n = 826) = 8.171, p = 0.004, with non-CVA patients scoring lower FES scores (Table 6).

**Table 6:** Association of CVA diagnosis and MFS and FES group (n=2073). CVA: Cerebral Vascular Accident; MFS: Morse Fall Score; FES: Fall Efficacy Scale.

Measure	*X *^2^	df	Sig. (2-tailed)
CVA diagnosis with MFS group	15.843	2	<0.001***
CVA diagnosis with FES group	8.171	1	0.004**

However, when analyzing the MFS and FES as scalar data and not groups, only MFS showed statistically significant differences among patients with and without a CVA diagnosis, t (2071) = -3.348, p < 0.001, indicating patients with a CVA diagnosis scored higher MFS than patients without a CVA diagnosis (Table 7). The expected differences in fall efficacy associated with a higher risk of falling were not consistently demonstrated within this sample across all groups.

**Table 7:** Independent sample t-tests for MFS, FES scores, and CVA diagnosis (n=2,074). **p < 0.01. CVA: Cerebral Vascular Accident; MFS: Morse Fall Score; FES: Fall Efficacy Scale.

Measure	t	df			Sig. (2-tailed)	Mean difference	95% CI of the difference	
							Lower	Upper
MFS	-3.348		2071	0.001**		-4.035	-6.399	-1.672
FES	-1.277		828	0.202		-0.853	-2.164	0.458

FES risk groups and age over 65 were significant predictors of a fall when patients with a primary CVA diagnosis were omitted from the sample (p = 0.022 and 0.046 respectively). Specifically, logistic regression was used to analyze the relationship between fall efficacy groups (high vs. low), age ≥ 65 years, and the occurrence of an in-hospital fall. It was found that, holding the other predictor variable constant, the probability of an in-hospital fall occurring had an OR of 5.632 (95% CI (2.171-7.892)) for a one-unit increase in the fall efficacy score (0 = low; 1 = high). When holding the fall efficacy score predictor variable constant, the odds of an in-hospital fall occurring had an OR of 1.21 (95% CI (1.006-1.442)) for a one-unit increase in age ≥ 65 years (0 = < 65 years of age; 1 = ≥ 65 years) (Table 8). The findings demonstrated an association of higher efficacy to not fall and increased age with an increased fall risk within this sample.

**Table 8:** Logistic regression analysis of variables associated with in-hospital falls.

Variables	Adjusted OR	95% CI	P-value
Intercept	0.002	0.001-0.051	0.066
Fall Efficacy Score High	5.632	2.171-7.892	0.022
Age => 65 Years	1.21	1.006, 1.442	0.046

## Discussion

Main findings

Sample and Instrument Reliability

This study found that the Short-FES-I and the MORSE demonstrated good reliability in this sample, consistent with prior literature findings [3]. There was less than 1% missing data for the project period, which provided a large sample size for comparison across the pre- and post-implementation periods. These findings strengthen the internal validity of the study as inferences were not limited by missing data. It is important to note that the population in our study was a relatively heterogeneous sample with age dispersion. However, the sample was limited to an in-patient adult population admitted to a medical unit, which limits the heterogeneity of the sample for setting and diagnosis. This may account for the high reliability estimates and warrants consideration when interpreting the estimates.

Aims of the Study

Interestingly, the specific patient subpopulations hypothesized to be at a higher risk of falls due to a false sense of self-efficacy regarding fall prevention were not consistently demonstrated within this sample. Males, as expected, did demonstrate more efficacy in fall prevention compared to females. However, patients with a CVA diagnosis or CVA-like symptoms on admission demonstrated a significantly less efficacious score in preventing falls compared to non-CVA patients. In contrast, prior studies show that stroke patients overestimated their abilities (despite fall prevention education) in the hospital setting and were unaware that their risk for falls could change with their medical condition [4]. Although it is known that falls efficacy moderately correlates with activities of daily living performance, rehabilitation for individuals after a stroke rarely concentrates on self-efficacy, with priority focused on the improvement of physical and mental function [14]. Further, as patients' FES risk grouping and age over 65 were significant predictors of a fall when patients with a primary CVA diagnosis were excluded, these findings support further exploration of including additional variables beyond clinical measures in fall risk assessment. The null findings regarding males with higher self-efficacy to prevent falls compared to females in this sample may be explained by consistency with prior research findings. Gazibara T et al. (2017) found that females have a higher degree of activity restrictions, indoor falling, and falling in general, regardless of location [15]. Gettens S and Fulbrook P (2015) also found that males from an in-hospital sample rated themselves more confident than females on both admission and discharge [17]. Our finding that patients with a CVA diagnosis or CVA-like symptoms on admission had a significantly less efficacious score to prevent falls compared to non-CVA patients is consistent with some prior studies. Park EY et al. (2018) found low FES scores in a post-CVA hemiplegic population [14]. However, their sample consisted of community-dwelling patients whose average time since their CVAs was nine years, compared to our sample of patients acutely admitted for a CVA or CVA symptoms. Dabkowski E et al. (2022) in a scoping review of the literature that included 41 studies, found patients who had a stroke were unaware that their risk for falls could change post-CVA and had a false sense of efficacy regarding falling [4]. Our findings that patients' FES risk grouping and age over 65 were significant predictors of a fall for patients without a CVA diagnosis are consistent with Gettens S and Fulbrook P (2015) findings in a similar population [17]. Whereas Greenberg M et al. (2021) did not find FES scores to be a significant predictor of falls [16]. This may be explained as their sample, in contrast to our hospitalized participants, were patients living in the community who self-reported falls during monthly follow-up phone calls after a visit to the Emergency Department for a fall. Additionally, Kuhlenschmidt ML et al. (2016) had similar findings that patients who reported feeling efficacious they would not fall were the group that experienced the most falls [18]. Like our study, their patients were adult patients who were acutely hospitalized. However, Kuhlenschmidt ML et al.’s (2016) sample was of patients on a bone marrow transplant unit, not a medical unit.

Clinical Significance and Sustainability

Falls in the inpatient setting result in significant physical and economic burdens to patients, including increased injury and mortality rates. This extends to the medical organization as well, causing burdens such as increased lengths of stay, medical care costs, and litigation [1]. Although universal fall precautions remain in place for all patients (regardless of the risk of falling), prior research shows that individualized interventions based on a patient’s risk factors are more likely to reduce falls than universal fall precautions [2]. In addition to examining physical risk factors for falls, it is advisable that clinicians also take into account other important factors like fall self-efficacy and how it impacts a patient’s risk for falls. The use of the Short FES-I allowed for increased staff awareness and increased motivation for continued performance improvement to ensure that patients remain in a safe environment. The clinical significance of maintaining the project methodology and momentum was to achieve sustainable change. Understanding patients’ perspectives and understanding of their fall risk can inform fall prevention policies in hospital settings [4]. Utilizing specific sustainment strategies with evidence-based interventions will allow for long-term change and foster a culture of evidence-based practice [26].

Strengths and weaknesses of this study

Within our study, there was less than 1% missing data, which provided a large sample and strengthened the internal validity of the study. Additionally, our large sample size allowed for predictive modeling of the probability of falls using the variables we found to be associated with falls in our exploratory analysis. Threats to internal validity such as self-selection and instrumentation were controlled for, with all patients meeting inclusion criteria being included in the study and rater training and assessing for inter-rater reliability estimates. Further, our study measured a fall as an observed fall, thereby increasing the internal validity of our findings [27].

Limitations

There were limitations in our study. Our study was a retrospective design using data originally not collected for research, but rather for a quality project. This limited the variables available for a multivariate analysis and the testing of other possible factors associated with a fall. Additionally, there were limitations in the generalizability of our study findings as our sample was limited to acutely hospitalized adults on a medical unit who had the cognitive ability to answer the questions within the Short FES-I. Another limitation of our study was the interruption of the study and data collection due to the COVID-19 pandemic’s strain on nursing resources, which is a historical threat to internal validity, and had the potential to impact the sustainability of the intervention during the second post-implementation period of 24 weeks. Further, our baseline testing could introduce a testing threat to the internal validity of our study. Also, the lack of a comparison group and the delivery of an educational intervention to reduce falls also threaten the internal validity of the study’s findings. Lastly, the lack of random assignment to treatment could not be controlled for with the study design; this limits the internal and external validity of our findings.

## Conclusions

The findings suggest that efficacy screening may be associated with decreased falls in acute care non-CVA inpatient populations over 65 years of age. Further research into the predictive utility of fall efficacy screening in acute care CVA and non-CVA hospitalized patient populations aged 65 years and above is recommended. Additionally, future research should include the evaluation of other possible confounding factors, in addition to clinical and physiological ones, in fall risk assessment.
